# Clinicopathological characteristics and prognosis of early-onset colorectal cancer: a retrospective cohort study

**DOI:** 10.3389/fonc.2026.1788135

**Published:** 2026-04-27

**Authors:** Shuping Lian, Guibin Zheng, Yixin Lin, Xun Fang, Yincong Guo, Jing Ma

**Affiliations:** 1 Department of Colorectal and Anal Surgery, Zhangzhou Affiliated Hospital of Fujian Medical University, Zhangzhou, China; 2 Department of Proctology, Zhangzhou Affiliated Hospital of Fujian Medical University, Zhangzhou, China

**Keywords:** clinicopathological characteristics, early-onset colorectal cancer, overall survival, prognosis, propensity score matching

## Abstract

**Objective:**

To investigate the clinical and pathological characteristics of patients with early-onset colorectal cancer (EOCRC) and to evaluate their impact on postoperative overall survival (OS) within the observed follow-up period.

**Methods:**

Consecutive colorectal cancer patients who underwent radical surgery at Zhangzhou Affiliated Hospital of Fujian Medical University between 2017 and 2024 were included. Patients were categorised into EOCRC group (≤50 years), and late-onset colorectal cancer (LOCRC) group (>50 years) based on age at diagnosis. To reduce baseline confounding, 1:4 propensity score matching (PSM) was performed. Stratified survival analyses were conducted across different TNM staging levels.

**Results:**

A total of 4,596 colorectal cancer patients were included, including 590 cases of EOCRC and 4,006 cases of LOCRC. Prior to PSM, EOCRC patients showed a higher proportion of females, lower ASA staging, increased prevalence of poorly differentiated tumours, mucinous adenocarcinoma/signet ring cell carcinoma, and dMMR status, alongside a relatively more advanced stage distribution. Median follow-up duration was 34.5 months. Both in the unmatched and matched cohorts, EOCRC patients demonstrated significantly superior OS compared to LOCRC patients. Multivariate Cox regression analysis demonstrated that EOCRC was an independent protective factor for OS both before (HR = 0.650, 95% CI 0.520–0.814) and after matching (HR = 0.641, 95% CI 0.509–0.809). Further stratified analysis by disease stage indicated that, among patients with stages I–IV disease, EOCRC demonstrated non-inferior OS outcomes compared with LOCRC, with the survival advantage being more pronounced in stage II and stage III patients.

**Conclusion:**

In a large single-centre retrospective cohort, despite presenting with certain unfavourable clinical and pathological characteristics at diagnosis, patients with EOCRC demonstrated OS comparable to those with LOCRC, exhibiting superior survival trends in certain staging groups. Prognosis assessment and clinical management of colorectal cancer should not rely solely on age at onset, but rather involve comprehensive decision-making incorporating staging, pathological features, and the patient’s overall condition.

## 1 Introduction

Colorectal cancer (CRC) remains one of the foremost cancer burdens globally. According to the latest global cancer statistics, CRC ranks as the third most commonly diagnosed malignancy and the second leading cause of cancer-related death worldwide, accounting for more than 1.9 million new cases and approximately 930,000 deaths annually (1). Recently, the rising incidence of EOCRC, defined as CRC diagnosed at ≤50 years of age, has become a global phenomenon. Multiple population-based studies have confirmed these increasing incidence patterns across different regions, particularly in North America and Asia (2–4). In addition, birth cohort effects and regional variations have also been reported in several countries, including China (5). Collectively, these epidemiological trends have positioned EOCRC as an increasingly important focus in contemporary CRC research and clinical practice.

Previous studies generally recognise differences in the clinical and pathological characteristics between EOCRC and late-onset colorectal cancer (LOCRC): EOCRC patients are more likely to present with poorly differentiated tumours and distinct histological subtypes, including mucinous adenocarcinoma and signet ring cell carcinoma (6, 7). These features are commonly considered indicative of a more aggressive biological behaviour. However, whether the long-term prognosis of EOCRC is worse, better, or comparable to that of LOCRC remains inconclusive (7–12). A meta-analysis published in 2025 indicated that although EOCRC is more commonly diagnosed at later stages, this difference does not necessarily translate into poorer tumour-specific survival outcomes. Furthermore, existing studies have reported inconsistent findings regarding survival outcomes in EOCRC (8). Studies utilising large-scale databases indicate that long-term survival comparisons between EOCRC and LOCRC may vary across different age reference groups, outcome measures, and statistical adjustment strategies (10). Rather than reflecting methodological inadequacy, the discordant findings across studies are more likely attributable to variations in population structure, endpoint selection, and analytical strategies.

Given the aforementioned background, this study relies on a single-centre, prospectively collected surgical cohort (2017–2024) to systematically compare the clinical and pathological characteristics of patients with EOCRC and LOCRC. OS serves as the primary endpoint to evaluate the independent association between age and prognosis. To mitigate confounding bias from baseline differences, this study further employed 1:4 propensity score matching (PSM) combined with multivariable Cox regression analyses. Stratified survival comparisons were conducted across different TNM stages, aiming to provide more reliable real-world evidence for risk stratification and follow-up management in EOCRC.

## 2 Methods

### 2.1 Study design

A retrospective analysis was conducted including consecutive CRC patients diagnosed and treated in the Department of Colorectal and Anal Surgery at Zhangzhou Affiliated Hospital of Fujian Medical University between January 2017 and December 2024. Clinical data, surgical records, pathological findings, and follow-up information were extracted from the hospital electronic medical record system by two independent researchers who cross-checked their work to ensure data integrity and accuracy.

### 2.2 Inclusion and exclusion criteria

Patients meeting the following criteria were included: ① Postoperative pathology confirming a diagnosis of colorectal adenocarcinoma; ② Underwent surgical resection with curative intent, via laparoscopic or open surgery; ③ Surgical resection met the principles of radical resection; ④ Possession of complete, traceable clinical records, surgical documentation, and postoperative pathological findings; ⑤ Availability of definitive follow-up information and survival outcomes; ⑥ Postoperative pathological staging based on the 8th edition of the American Joint Committee on Cancer (AJCC) TNM staging system.

**Figure 1:**
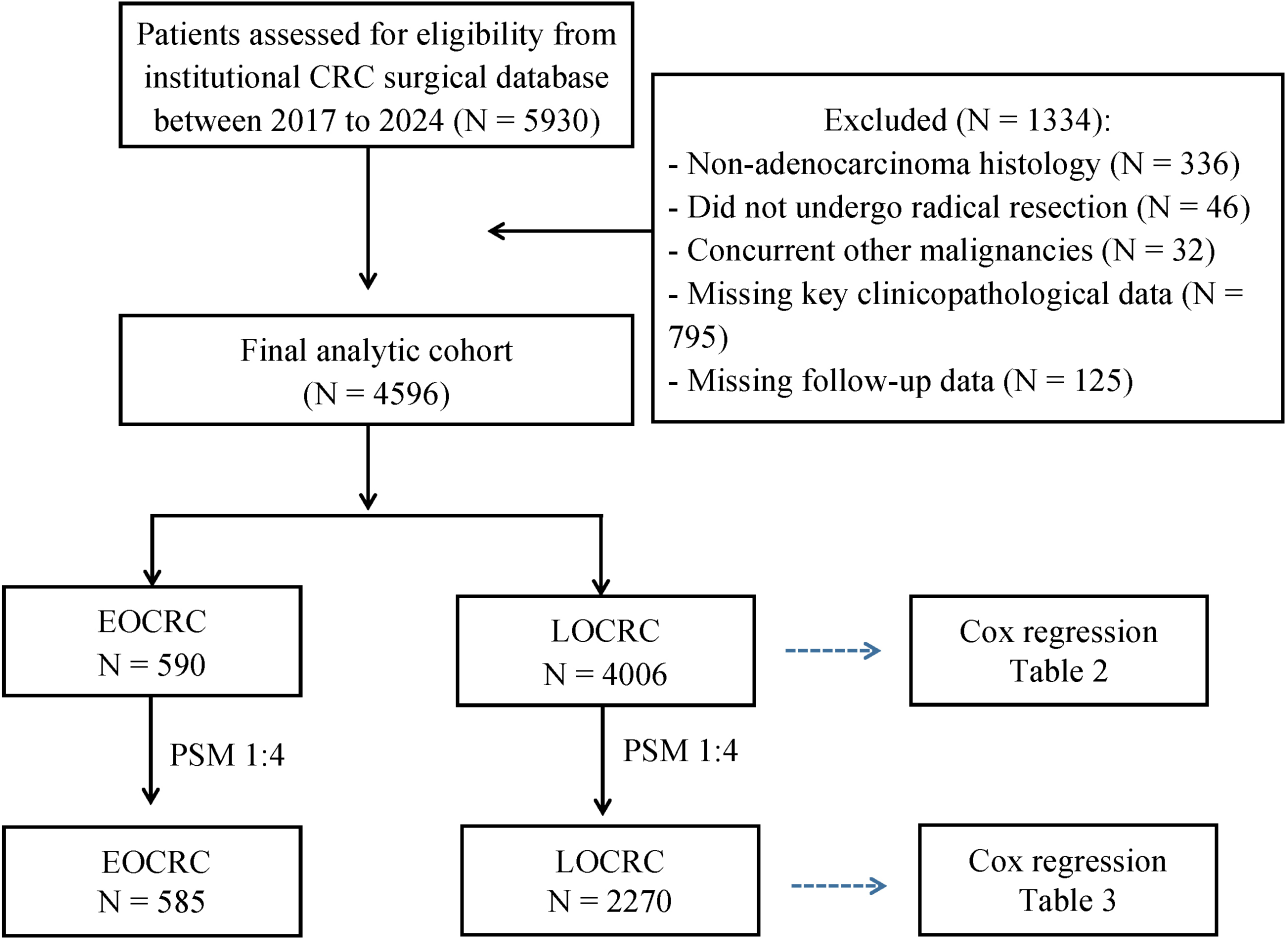
Study flowchart.

Patients meeting any of the following criteria were excluded: ① Unable to undergo surgical treatment due to extensive tumour metastasis, systemic intolerance, or other medical reasons; ② Initially assessed as unresectable, failing to meet surgical criteria after conversion therapy (e.g., neoadjuvant chemotherapy or chemoradiotherapy), and ultimately not undergoing radical resection; ③ Underwent solely palliative or exploratory surgery; ④ Concurrent malignancies at other sites potentially confounding survival outcome assessment; ⑤ Other histological subtypes such as neuroendocrine tumours, lymphomas, and sarcomas were excluded due to their distinct biological behaviour, treatment strategies, and prognostic patterns, which could introduce substantial heterogeneity into survival analyses. ⑥ Missing key clinical variables, pathological data, or follow-up information precluding valid statistical analyses. The patient selection process, including the numbers of screened, excluded, and finally included patients, as well as the number lost to follow-up, is summarised in the study flowchart (**Figure 1**).

### 2.3 Definition of age groups

Patients were grouped according to age at diagnosis, defined as the actual age at the time of the first definitive pathological diagnosis of CRC:

EOCRC is defined as diagnosis at ≤50 years of age; LOCRC is defined as diagnosis at >50 years of age (10).

### 2.4 Treatment principles and overall strategy

All treatment decisions for included patients were made within a unified diagnostic and therapeutic framework, adhering to CRC guidelines, including those of the National Comprehensive Cancer Network^®^ (NCCN^®^) and the Chinese Society of Clinical Oncology^®^ (CSCO^®^). Treatment strategies were primarily determined based on tumour staging, pathological characteristics, and the patient’s overall condition.

Surgical treatment: All patients underwent radical surgery, with the approach (laparoscopic or open) selected based on tumour location, stage, and patient condition. For colon cancer patients, regional lymph node dissection was performed adhering to the principle of ligating the tumour’s feeding vessels at their origin. For rectal cancer patients, the procedure followed the principle of total mesorectal excision (TME). Surgical management was conducted within a standardized institutional framework by a dedicated colorectal surgical team, adhering to consistent oncological principles throughout the study period. Decisions regarding surgical approach were guided by tumour characteristics and established clinical guidelines, thereby minimizing practice variation.

Postoperative adjuvant treatment strategies: Indications and regimens for postoperative adjuvant therapy were primarily determined based on pathological staging and high-risk factors. Generally, stage I patients did not require further treatment post-surgery; stage II patients with high-risk pathological features (such as poorly differentiated tumours, lymphovascular invasion or perineural invasion) should be considered for adjuvant chemotherapy; stage III patients should, in principle, receive postoperative adjuvant chemotherapy. Common regimens included fluorouracil-based chemotherapy (e.g., FOLFOX, CAPOX). For stage IV CRC patients, postoperative treatment strategies were determined by a multidisciplinary team (MDT) following comprehensive assessment of metastatic distribution, resectability, tumour burden, and overall patient condition. Specific regimen selection is decided by the MDT after considering patient tolerance. Postoperative monitoring follows international guideline recommendations (13). Patients underwent assessment every three months for the first three years, every six months for years four to five, and annually thereafter. Follow-up is conducted via outpatient clinics, mail, or telephone.

### 2.5 Primary endpoint

The primary endpoint was the impact of age on overall survival (OS). OS was defined as the time from the date of surgery to death from any cause or the date of last follow-up. Patients still alive at the end of follow-up were censored at the date of last follow-up. The date of last follow-up was June 2025. Secondary endpoints included: differences in clinical and pathological characteristics between age groups; and differences in OS across different TNM staging levels within each age group.

### 2.6 Statistical analyses

Continuous variables were presented as mean ± standard (SD) deviation according to their distribution, with intergroup comparisons assessed using Student’s t-test. Categorical variables were presented as counts and percentages, with intergroup comparisons performed using the chi-square test. Kaplan–Meier survival curves were constructed using R software (R Foundation for Statistical Computing, Vienna, Austria), and survival differences between age groups were compared using the log-rank test. Cox proportional hazards regression models were employed for univariate and multivariate analyses to assess the independent association between age grouping and OS.

To further examine the association between age and prognosis and minimise confounding bias arising from baseline differences, PSM was employed to achieve intergroup balance. PSM was calculated via a logistic regression model incorporating the following covariates: sex, body mass index (BMI), American Society of Anaesthesiologists (ASA) grade, tumour location, carcinoembryonic antigen (CEA), carbohydrate antigen 19-9 (CA19-9), tumour differentiation grade, histological type, perineural invasion, lymphovascular invasion, tumour deposit, pT stage, and pN stage. A 1:4 nearest neighbour matching approach was employed with a propensity score cutoff of 0.2 (logit of propensity score). Post-matching, covariate balance was assessed using standardised mean differences (SMD), with SMD < 0.10 indicating satisfactory balance.

All statistical analyses were performed using SPSS 29 (IBM SPSS Statistics, Chicago, IL, USA), with survival curves plotted using R software. All statistical tests were two-tailed, and a P value < 0.05 was considered statistically significant.

## 3 Results

### 3.1 Patient characteristics

**Figure 2:**
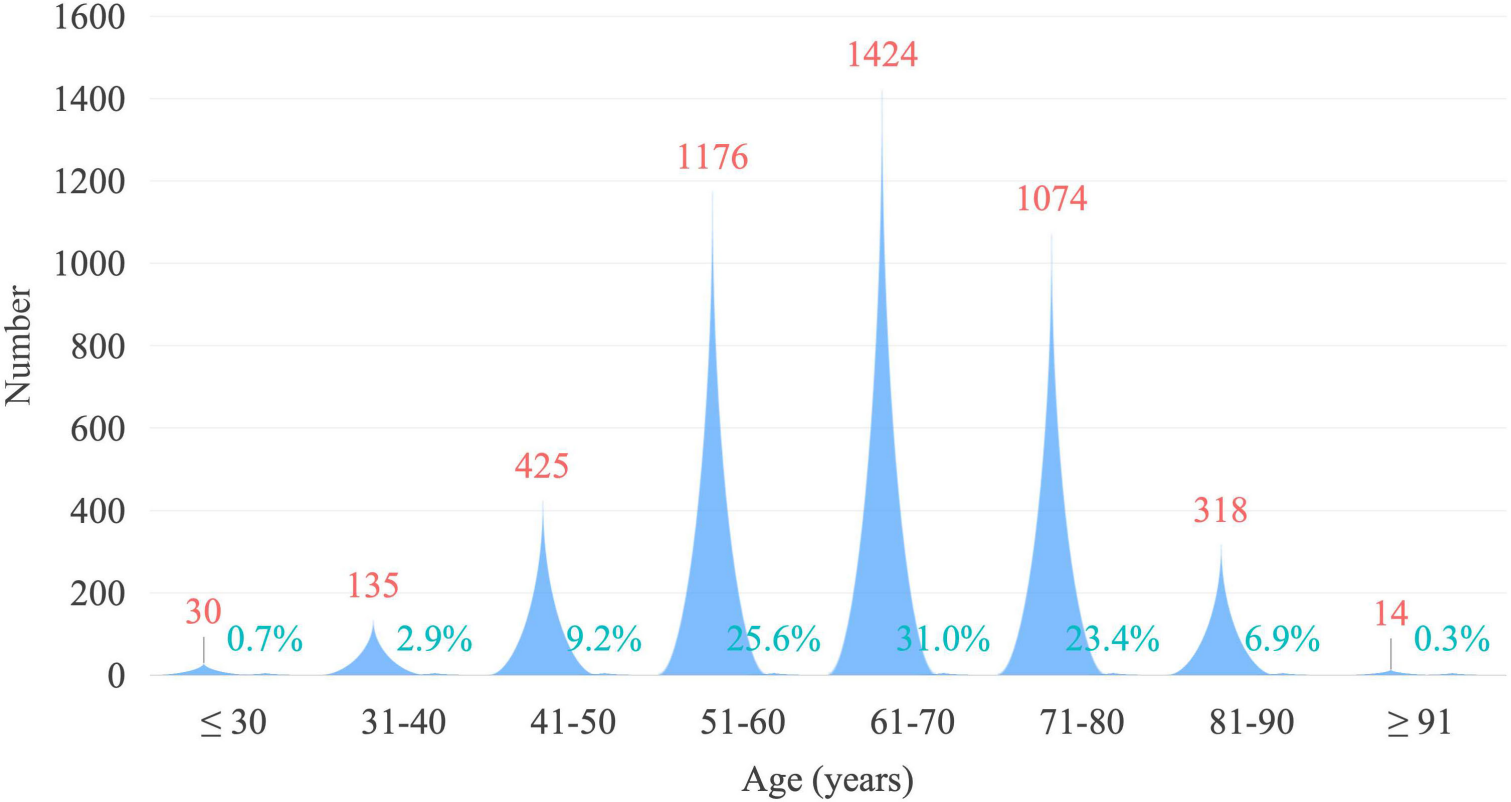
Age distribution of patients with colorectal cancer.

During the study period, a total of 5,930 patients were initially screened from the institutional database, of whom 125 were lost to follow-up. After excluding patients who did not meet the predefined eligibility criteria, 4,596 patients were finally included in the analytic cohort (**Figure 1**). Among these, 590 cases (12.8%) were classified as EOCRC and 4,006 cases (87.2%) as LOCRC. The age at diagnosis for the overall cohort was predominantly concentrated between 51 and 80 years, with the highest number of patients in the 61–70 age group, followed by those aged 51–60 and 71–80. A significant proportion of patients were aged ≤50 years, whilst those aged ≥80 years constituted a relatively smaller proportion (**Figure 2**).

### 3.2 Comparison of clinical and pathological characteristics before PSM

Before PSM, significant differences were observed between EOCRC and LOCRC patients (**Table 1**). Compared with the LOCRC group, the EOCRC group had a higher proportion of female patients (46.9% vs. 39.8%, P<0.001) and a markedly higher proportion of ASA 1–2 status (82.0% vs. 65.7%, P<0.001). Pathologically, EOCRC patients more frequently presented with poorly differentiated tumours (26.6% vs. 21.4%, P = 0.004) and mucinous adenocarcinoma or signet ring cell carcinoma (15.8% vs. 11.0%, P<0.001). The prevalence of deficient mismatch repair (dMMR) was also significantly higher in EOCRC patients (9.2% vs. 5.9%, P = 0.002). In terms of nodal involvement and stage distribution, EOCRC patients had a higher proportion of pN2 disease (28.0% vs. 20.7%, P<0.001) and stage IV tumours (15.9% vs. 13.1%, P = 0.014), whereas elevated preoperative CEA levels were less frequent in EOCRC patients (33.2% vs. 42.0%, P<0.001).

**Table 1:** Patients’ clinical and pathological characteristics before and after propensity score matching.

Variables	Before PSM: LOCRC (n = 4006)	Before PSM: EOCRC (n = 590)	Before PSM: P value	After PSM: LOCRC (n = 2270)	After PSM: EOCRC (n = 585)	After PSM: P value
Sex			<0.001			0.592
Female	1595 (39.8)	277 (46.9)		1039 (45.8)	275 (47.0)	
Male	2411 (60.2)	313 (53.1)		1231 (54.2)	310 (53.0)	
Body mass index (kg/m^2^)			0.084			0.763
≤25	3257 (81.3)	462 (78.3)		1794 (79.0)	459 (78.5)	
>25	749 (18.7)	128 (21.7)		476 (21.0)	126 (21.5)	
ASA grade			<0.001			0.615
1~2	2630 (65.7)	484 (82.0)		1838 (81.0)	479 (81.9)	
3~5	1376 (34.3)	106 (18.0)		432 (19.0)	106 (18.1)	
Perforation before surgery			0.774			0.972
No	3833 (95.7)	563 (95.4)		2166 (95.4)	558 (95.4)	
Yes	173 (4.3)	27 (4.6)		104 (4.6)	27 (4.6)	
Obstruction before surgery			0.441			0.601
No	3851 (96.1)	571 (96.8)		2186 (96.3)	566 (96.8)	
Yes	155 (3.9)	19 (3.2)		84 (3.7)	19 (3.2)	
Tumour location			0.001			0.443
Rectum	1997 (49.9)	252 (42.7)		1018 (44.8)	252 (43.1)	
Colon	2009 (50.1)	338 (57.3)		1252 (55.2)	333 (56.9)	
Tumour sidedness			0.132			0.406
Left-sided	3255 (81.3)	464 (78.6)		1820 (80.2)	460 (78.6)	
Right-sided	751 (18.7)	126 (21.4)		450 (19.8)	125 (21.4)	
CEA (mg/ml)			<0.001			0.758
<5	2325 (58.0)	394 (66.8)		1498 (66.0)	390 (66.7)	
≥5	1681 (42.0)	196 (33.2)		772 (34.0)	195 (33.3)	
CA19-9 (U/ml)			0.197			0.624
<37	3286 (82.0)	471 (79.8)		1844 (81.2)	470 (80.3)	
≥37	720 (18.0)	119 (20.2)		426 (18.8)	115 (19.7)	
Perineural invasion			0.969			0.826
Negative	1063 (26.5)	157 (26.6)		599 (26.4)	157 (28)	
Positive	2943 (73.5)	433 (73.4)		1671 (73.6)	428 (73.2)	
Lymphovascular invasion			0.227			0.780
Negative	1783 (44.5)	247 (41.9)		973 (42.9)	247 (42.2)	
Positive	2223 (55.5)	343 (58.1)		1297 (57.1)	338 (57.8)	
MMR status			0.002			0.618
pMMR	3770 (94.1)	536 (90.8)		2079 (91.6)	532 (90.9)	
dMMR	236 (5.9)	54 (9.2)		191 (8.4)	53 (9.1)	
Tumour differentiation grade			0.004			0.587
Poorly	857 (21.4)	157 (26.6)		565 (24.9)	152 (26.0)	
Well-Moderately	3149 (78.6)	433 (73.4)		1705 (75.1)	433 (74.0)	
Histopathology			<0.001			0.905
Adenocarcinoma	3567 (89.0)	497 (84.2)		1933 (85.2)	497 (85.0)	
Mucinous adenocarcinoma/Signet-ring cell	439 (11.0)	93 (15.8)		337 (14.8)	88 (15.0)	
Tumour deposit			0.618			0.705
Negative	3710 (92.6)	543 (92.0)		2102 (92.6)	539 (92.1)	
Positive	296 (7.4)	47 (8.0)		168 (7.4)	46 (7.9)	
pT stage			0.585			0.825
T0~3	2322 (58.0)	349 (59.2)		1354 (59.6)	346 (59.1)	
T4	1684 (42.0)	241 (40.8)		916 (40.4)	239 (40.9)	
pN stage			<0.001			0.875
N0	2024 (50.5)	269 (45.6)		1067 (47.0)	269 (46.0)	
N1	1152 (28.8)	156 (26.4)		604 (26.6)	156 (26.7)	
N2	830 (20.7)	165 (28.0)		599 (26.4)	160 (27.4)	
pTNM stage			0.014			0.95
0~I	556 (13.9)	94 (15.9)		359 (15.8)	94 (16.1)	
II	1358 (33.9)	164 (27.8)		662 (29.2)	164 (28.0)	
III	1567 (39.1)	238 (40.3)		910 (40.1)	236 (40.3)	
IV	525 (13.1)	94 (15.9)		339 (14.9)	91 (15.6)	

LOCRC, Late-onset colorectal cancer; EOCRC, Early-onset colorectal cancer; CEA, carcinoembryonic antigen; CA19-9, carbohydrate antigen 19-9; MMR, mismatch repair; ASA, American Society of Anaesthesiologists.

### 3.3 Comparison of clinical and pathological characteristics after PSM

After PSM, a total of 585 EOCRC and 2270 LOCRC were included. After matching, no statistically significant differences were observed between the two groups in key clinical and pathological characteristics including sex, BMI, ASA classification, tumour location, CEA, CA19-9, tumour differentiation grade, histological type, lymphovascular invasion, perineural invasion, tumour deposit, or pT, pN and TNM staging (all P > 0.05), indicating well-balanced baseline characteristics (**Table 1**).

### 3.4 OS and Cox regression analyses before PSM

The median follow-up time was 34.5 (range, 1–104) months.

**Figure 3:**
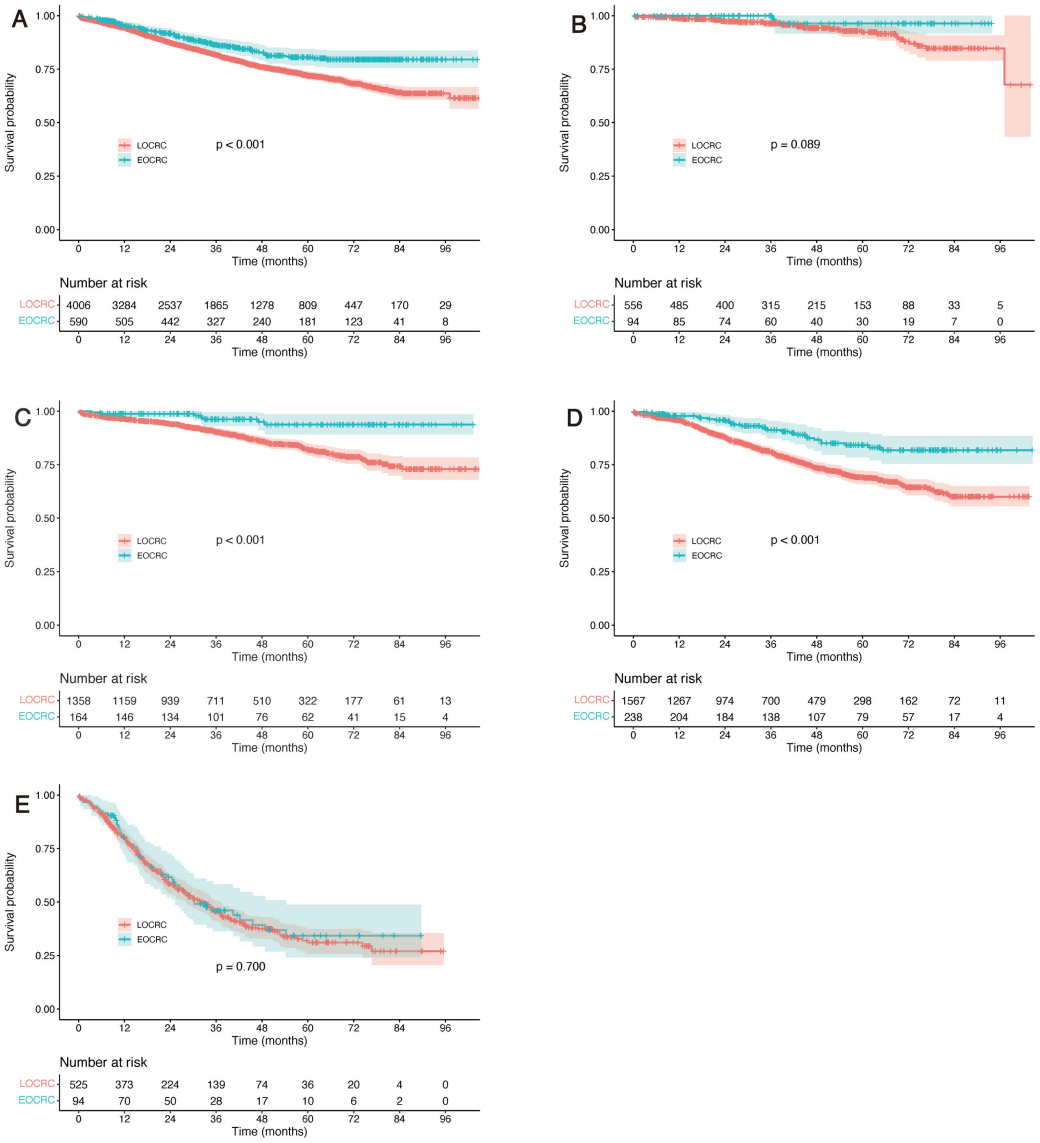
Overall survival curves for early-onset versus late-onset colorectal cancer patients before propensity score matching. **(A)** Overall survival curve for the entire cohort; **(B-E)** Overall survival curves for patients with TNM stage I, II, III, and IV disease respectively.

Kaplan–Meier survival analysis demonstrated significantly better OS in EOCRC patients compared with LOCRC patients (P value’s log-rank test < 0.001). The estimated 3-year OS rate was 86.4% in the EOCRC group versus 81.7% in the LOCRC group, suggesting that the survival advantage was most pronounced within the first three years after surgery, which is consistent with the median follow-up duration of this study (**Figure 3A**).

In the multivariable Cox regression analysis, compared with LOCRC, patients with EOCRC demonstrated a significantly reduced risk of death (HR = 0.650, 95% CI 0.520–0.814, P<0.001), confirming EOCRC as an independent protective factor for OS within the observed follow-up period. In the overall unmatched cohort, ASA grade 3–5 (HR = 1.945, 95% CI 1.692–2.235), elevated CA19–9 levels (HR = 1.809, 95% CI 1.556–2.102), positive tumour deposits (HR = 1.646, 95% CI 1.349–2.008), T4 stage (HR = 1.440, 95% CI 1.251–1.657), and N2 stage (HR = 1.604, 95% CI 1.458–1.764) were independently associated with worse OS, irrespective of age group. These factors remained significant predictors of adverse outcomes after adjustment for EOCRC versus LOCRC status (**Table 2**).

**Table 2:** Univariate and multivariate Cox regression analyses of factors associated with OS before propensity score matching.

Variables	Univariate analysis HR		Multivariate analysis HR	
	HR (95% CI)	P	HR (95% CI)	P
EOCRC vs. LOCRC	0.638 (0.510~0.799)	<0.001	0.650 (0.520~0.814)	<0.001
Sex (Man vs. Female)	1.098 (0.958~1.259)	0.180		
BMI (>25 vs. ≤25 kg/m^2^)	0.859 (0.717~1.029)	0.099		
ASA grade (1~2 vs. 3~5)	1.903 (1.644~2.201)	<0.001	1.945 (1.692~2.235)	<0.001
Perforation before surgery (Yes vs. No)	1.159 (0.891~1.509)	0.271		
Obstruction before surgery (Yes vs. No)	1.280 (0.993~1.651)	0.057	1.294 (1.005~1.664)	0.045
Tumour location (Colon vs. Rectum)	1.048 (0.897~1.224)	0.557		
Tumour sidedness (Right-sided vs. Left-sided)	1.196 (0.993~1.439)	0.059	1.251 (1.065~1.470)	0.006
CEA (>5 vs. ≤5 mg/ml)	1.296 (1.122~1.496)	<0.001	1.301 (1.127~1.501)	<0.001
CA19-9 (>37 vs. ≤37 U/ml)	1.806 (1.553~2.100)	<0.001	1.809 (1.556~2.102)	<0.001
Perineural invasion (Positive vs. Negative)	1.068 (0.888~1.284)	0.486		
Lymphovascular invasion (Positive vs. Negative)	1.271 (1.071~1.507)	0.006	1.290 (1.091~1.525)	0.003
MMR status (dMMR vs. pMMR)	0.505 (0.348~0.735)	<0.001	0.516 (0.355~0.749)	<0.001
Tumour differentiation grade (Well-Moderately vs. Poorly)	0.831 (0.708~0.975)	0.023	0.823 (0.704~0.961)	0.014
Histopathology (Mucinous adenocarcinoma/Signet-ring cell vs. Adenocarcinoma)	1.026 (0.836~1.260)	0.806		
Tumour deposit (Positive vs. Negative)	1.615 (1.323~1.973)	<0.001	1.646 (1.349~2.008)	<0.001
pT (T4 vs. T0~3)	1.416 (1.228~1.632)	<0.001	1.440 (1.251~1.657)	<0.001
pN (N2 vs. N1, N0)	1.697 (1.460~1.770)	<0.001	1.604 (1.458~1.764)	<0.001

LOCRC, Late-onset colorectal cancer; EOCRC, Early-onset colorectal cancer; CEA, carcinoembryonic antigen; CA19-9, carbohydrate antigen 19-9; MMR, mismatch repair; ASA, American Society of Anaesthesiologists.

At multivariable analysis, pTNM stage was not included to avoid structural multicollinearity, as it is derived from pT and pN. Total sample size: N = 4596.

### 3.5 OS and Cox regression analyses after PSM

**Figure 4:**
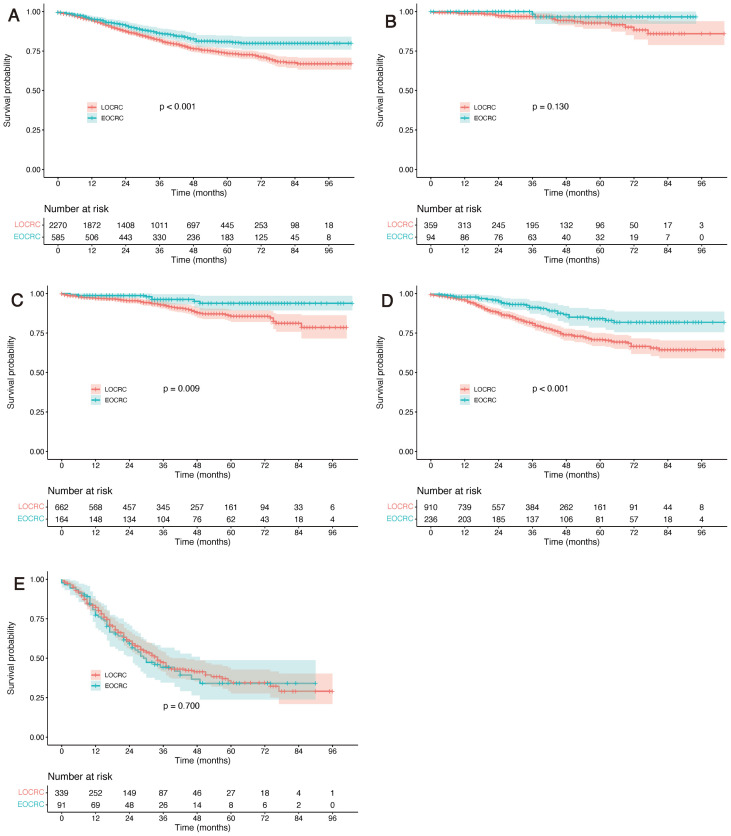
Overall survival curves for early-onset versus late-onset colorectal cancer patients after propensity score matching. **(A)** Overall survival curve for the entire cohort; **(B-E)** Overall survival curves for patients with TNM stage I, II, III, and IV disease respectively.

Within the matched cohort and during the same follow-up period (maximum 104 months), Kaplan–Meier survival curves continued to demonstrate superior OS in EOCRC patients compared with LOCRC patients (P value’s log-rank test < 0.001) (**Figure 4A**). In the matched cohort, multivariable Cox regression analysis demonstrated that EOCRC remained independently associated with a lower risk of mortality (HR = 0.641, 95% CI 0.509–0.809, P<0.001). ASA grade 3–5 (HR = 1.917, 95% CI 1.566–2.348), elevated CA19–9 levels (HR = 2.055, 95% CI 1.706–2.475), positive tumour deposits (HR = 1.606, 95% CI 1.243–2.075), T4 stage (HR = 1.633, 95% CI 1.352–1.974), and N2 stage (HR = 1.786, 95% CI 1.571–2.032) were independently associated with worse OS in the overall matched cohort, independent of age group (**Table 3**). These findings indicate that these clinicopathological factors serve as robust adverse prognostic indicators regardless of age at diagnosis, even after balancing baseline characteristics through PSM.

**Table 3:** Univariate and multivariate Cox regression analyses of factors associated with OS after propensity score matching.

Variables	Univariate analysis HR		Multivariate analysis HR	
	HR (95% CI)	P	HR (95% CI)	P
EOCRC vs. LOCRC	0.633 (0.501~0.799)	<0.001	0.641 (0.509~0.809)	<0.001
Sex (Man vs. Female)	1.000 (0.836~1.195)	0.998		
BMI (>25 vs. ≤25 kg/m^2^)	0.842 (0.670~1.060)	0.143		
ASA grade (1~2 vs. 3~5)	1.772 (1.417~2.214)	<0.001	1.917 (1.566~2.348)	<0.001
Perforation before surgery (Yes vs. No)	1.282 (0.919~1.787)	0.143		
Obstruction before surgery (Yes vs. No)	1.472 (1.049~2.066)	0.025	1.469 (1.049~2.056)	0.025
Tumour location (Colon vs. Rectum)	1.096 (0.891~1.347)	0.385		
Tumour sidedness (Right-sided vs. Left-sided)	1.292 (1.020~1.636)	0.034	1.361 (1.107~1.674)	0.003
CEA (>5 vs. ≤5 mg/ml)	1.237 (1.015~1.507)	0.035		
CA19-9 (>37 vs. ≤37 U/ml)	1.882 (1.532~2.311)	<0.001	2.055 (1.706~2.475)	<0.001
Perineural invasion (Positive vs. Negative)	0.84 (0.669~1.090)	0.205		
Lymphovascular invasion (Positive vs. Negative)	1.285 (1.014~1.628)	0.038	1.290 (1.024~1.625)	0.031
MMR status (dMMR vs. pMMR)	0.495 (0.321~0.763)	0.001	0.513 (0.334~0.789)	0.002
Tumour differentiation grade (Well-Moderately vs. Poorly)	0.818 (0.668~1.001)	0.051		
Histopathology (Mucinous adenocarcinoma/Signet-ring cell vs. Adenocarcinoma)	1.012 (0.793~1.293)	0.921		
Tumour deposit (Positive vs. Negative)	1.626 (1.257~2.104)	<0.001	1.606 (1.243~2.075)	<0.001
pT (T4 vs. T0~3)	1.581 (1.300~1.922)	<0.001	1.633 (1.352~1.974)	<0.001
pN (N2 vs. N1, N0)	1.776 (1.559~2.023)	<0.001	1.786 (1.571~2.032)	<0.001

LOCRC, Late-onset colorectal cancer; EOCRC, Early-onset colorectal cancer; CEA, carcinoembryonic antigen; CA19-9, carbohydrate antigen 19-9; MMR, mismatch repair; ASA, American Society of Anaesthesiologists.

At multivariable analysis, pTNM stage was not included to avoid structural multicollinearity, as it is derived from pT and pN. Total sample size: N = 2855.

### 3.6 Comparison of OS between EOCRC and LOCRC in the study cohort

Further stratified survival analysis was conducted according to TNM staging. Results demonstrated that in both before and after PSM cohorts, patients with EOCRC demonstrated comparable OS across TNM stages I–IV, with a tendency toward improved survival in certain stages. Notably, the survival advantage was more pronounced in patients with stage II and stage III disease (**Figures 3B–E**, **4B–E**).

## 4 Discussion

This large retrospective cohort study systematically evaluated the association between age at diagnosis and long-term survival outcomes in patients with CRC. Despite presenting with more advanced disease stages and several unfavourable clinicopathological characteristics, patients with early-onset EOCRC consistently demonstrated superior OS compared with those with LOCRC. Importantly, this association remained robust after multivariable adjustment and PSM, and persisted across different TNM staging strata. These findings suggest that age at diagnosis may function as an independent prognostic factor in CRC, rather than merely reflecting tumour aggressiveness or stage distribution.

EOCRC has traditionally been regarded as a biologically aggressive disease entity, characterised by poorer differentiation, higher rates of mucinous or signet ring cell histology, and more advanced stage at presentation (6, 7). However, evidence regarding long-term survival outcomes in EOCRC remains inconsistent. Several population-based studies using the Surveillance, Epidemiology, and End Results (SEER) Program and the National Cancer Database (NCDB) have reported inferior or comparable survival outcomes in EOCRC patients, particularly in analyses with limited adjustment for disease stage and treatment-related variables (10–12, 14, 15). In contrast, an increasing number of studies have demonstrated that, after adequate multivariable adjustment, EOCRC patients exhibit survival outcomes comparable to—or even superior to—those of LOCRC patients (11, 16). The present study aligns more closely with this latter body of evidence. Compared with prior investigations, this study provides strengthened methodological control over confounding factors through the combined use of multivariable Cox regression and 1:4 PSM. Baseline clinical and pathological characteristics were well balanced between EOCRC and LOCRC cohorts after matching, thereby reducing bias related to disease stage, tumour biology, and perioperative factors. Under these conditions, EOCRC remained a stable and independent protective factor for OS, supporting the notion that previously reported survival disadvantages in EOCRC may, at least in part, reflect residual confounding rather than true biological inferiority. These findings contribute high-quality real-world evidence from a large surgical cohort in China and help reconcile discrepancies observed in earlier studies.

Despite exhibiting a higher prevalence of aggressive clinicopathological features, EOCRC patients did not experience worse OS during the study follow-up period. This divergence between pathological presentation and survival outcome likely reflects a combination of host-, treatment-, and tumour-related factors. From a host-related perspective, younger patients typically have fewer comorbidities, lower ASA classifications, and better functional reserve, which translates into greater tolerance for radical surgery and perioperative management. Prior studies have shown that comorbidity burden and functional status exert prognostic effects on long-term survival that are comparable in magnitude to tumour stage itself (17, 18). This advantage allows EOCRC patients to more reliably complete standardised and intensive multimodal treatment. From a treatment-related standpoint, younger patients are generally more likely to receive guideline-concordant adjuvant therapy, tolerate dose-intensive regimens, and undergo salvage treatment following disease recurrence. Multiple studies have reported higher treatment completion rates and better access to subsequent therapies among younger CRC patients, which may partially explain their improved long-term survival outcomes (15, 19). Additionally, molecular heterogeneity may contribute to this phenomenon. Certain favourable molecular subtypes, such as dMMR, are relatively more prevalent in EOCRC, and dMMR status has consistently been associated with improved survival in CRC (20, 21). Taken together, these factors indicate that while EOCRC may display aggressive pathological features, modern comprehensive treatment strategies can effectively mitigate their adverse impact, allowing age-related advantages to become apparent after adequate confounding adjustment.

In stratified analyses, the survival advantage associated with EOCRC was not uniformly distributed across all stages but was most pronounced and consistent among patients with stage II and stage III disease. EOCRC patients are more frequently diagnosed at advanced stages and exhibit aggressive pathological features, suggesting that intermediate and locally advanced disease stages may represent a critical window during which age-related prognostic differences emerge (22). In clinical practice, management of stage II and III CRC often requires nuanced decision-making regarding adjuvant therapy, treatment intensity, and long-term follow-up, all of which may be influenced by patient age. Kanter et al. reported that EOCRC patients were more likely to receive intensified multidisciplinary treatment, with survival disparities becoming more evident in populations requiring systemic therapy (23). Our findings further support the hypothesis that age exerts a more discernible prognostic influence in disease stages characterised by higher tumour burden and greater treatment complexity. Notably, few prior studies have systematically evaluated age effects across different TNM stages, and this study provides clinically relevant evidence to inform stage-specific risk stratification and follow-up strategies in EOCRC.

Several limitations of this study should be acknowledged. First, as a retrospective single-centre analysis, residual confounding from unmeasured variables cannot be entirely excluded despite adjustment using multivariable Cox regression and PSM. Second, OS was used as the sole primary endpoint. Given that patients with EOCRC are generally younger and have a lower comorbidity burden, the observed survival advantage may partly reflect a reduced competing risk of non-cancer-related mortality rather than differences in tumour biology alone. Detailed cause-of-death information was not consistently available in this institutional database, precluding reliable cancer-specific survival analysis. Third, although surgical management was conducted within a standardized institutional framework, perioperative strategies and systemic treatment approaches may have evolved over the study period. Importantly, detailed treatment variables—including the rates and completion of neoadjuvant and adjuvant therapies—were not systematically analysed in this study. Finally, An additional limitation is that the present findings apply specifically to patients who met the predefined eligibility criteria, underwent curative-intent radical resection, and had available follow-up and survival data. Therefore, our conclusions should not be directly generalised to all patients with EOCRC or LOCRC, particularly those who were not surgical candidates, did not undergo radical resection, or were excluded during the screening process. Despite these limitations, this study has several notable strengths. It represents a large real-world surgical cohort with relatively consistent treatment principles and comprehensive clinicopathological data. The combined use of multivariable adjustment and PSM enhances methodological rigor and reduces baseline imbalance. Furthermore, stratified analyses across TNM stages provide clinically relevant insight into stage-specific age effects.

## 5 Conclusion

Despite more frequently presenting with certain unfavourable clinical and pathological characteristics at diagnosis, in this screened surgical cohort of patients underwent radical resection, patients with EOCRC exhibit no worse prognosis compared to those with LOCRC, demonstrating a superior survival trend particularly among stage II and III patients. Age at diagnosis is not a decisive factor for poor prognosis in CRC. Prognosis for patients with CRC should be determined through individualised decision-making that comprehensively considers tumour stage, pathological characteristics, and the patient’s overall condition. Future research should further validate the age effect within different stages across multicentre populations. This should be combined with molecular characteristics and treatment-related factors to continuously refine precise stratification and individualised management strategies for CRC patients of varying ages.

## Data Availability

The original contributions presented in the study are included in the article/supplementary material. Further inquiries can be directed to the corresponding authors.
